# Neuroleptic‐induced Parkinsonism: Clinicopathological study

**DOI:** 10.1002/mds.26467

**Published:** 2015-12-11

**Authors:** Umar A. Shuaib, Ali H. Rajput, Christopher A. Robinson, Alex Rajput

**Affiliations:** ^1^ Saskatchewan Movement Disorders ProgramRoyal University HospitalSaskatoonSaskatchewanCanada; ^2^ Division of Neurology, Saskatchewan Movement Disorders ProgramUniversity of Saskatchewan, Saskatoon Health RegionSaskatoonSaskatchewanCanada; ^3^ Neuropathology, Department of PathologyUniversity of Saskatchewan, Saskatoon Health RegionSaskatoonSaskatchewanCanada

**Keywords:** parkinsonism, drug induced, pathology, substantia nigra, neuroleptic‐induced

## Abstract

**Background:**

Drug‐induced parkinsonism is a well‐known complication of several different drugs—the most common being neuroleptic‐induced parkinsonism. However, very few autopsies have been reported in such cases.

**Methods:**

Patients assessed at Movement Disorders Clinic Saskatchewan are offered brain autopsy. Detailed clinical records are kept.

**Results:**

Brains were obtained from 7 drug‐induced parkinsonism patients with parkinsonian symptom onset coinciding with use of drugs known to produce parkinsonism. Six were on antipsychotics and 1 was on metoclopramide. Three cases were treated with levodopa for parkinsonism. In two cases, parkinsonian features reversed after stopping the offending agent. Both had autopsy evidence of preclinical PD. In 4 of the remaining 5, dopamine‐blocking drugs were continued until death. In 4 of those 5, brain histology revealed no cause for the parkinsonism, but 1 had mild SN neuronal loss without Lewy bodies.

**Conclusion:**

This study shows that reversal of parkinsonism after discontinuing offending drugs does not indicate absence of underlying pathology. Neuroleptics can unmask preclinical PD in patients with insufficient SN damage for the disease to manifest clinically. Though the mechanism of sustained parkinsonian features after discontinuing neuroleptics remains to be established, it is unlikely that dopamine receptor block leads to retrograde SN neuronal degeneration. Furthermore, l‐dopa does not appear to be toxic to SN. © 2015 International Parkinson and Movement Disorder Society

Drug‐induced parkinsonism (DIP) is defined as the appearance of parkinsonism on treatment with pharmaceutical agents. Most of those drugs impair dopamine (DA) function.^1^ DIP was first recognized in the early 1950s as a common complication of neuroleptic therapy—neuroleptic‐induced parkinsonism (NIP).^1^ Other drugs that produce DA receptor block or deplete DA^1^, ^2^ also produce parkinsonism. Initially, it was considered to be an exclusive side effect of first‐generation neuroleptics that block D2 receptors. Other agents, such as calcium channel blockers, gastrointestinal prokinetics, antiarrhythmics, and antidepressants, have also been implicated in DIP.^1^ The mechanisms of how these other drugs produce parkinsonism is not known.^1^

Older literature indicates that DIP is the second‐most common cause of parkinsonism.^3^, ^4^ The incidence of DIP may have declined with widespread use of atypical antipsychotics, but conclusive evidence is not available so far. In most cases, DIP is reversible after discontinuing the offending agent. However, in up to 25% of cases, symptoms persist after cessation of the drug.^5^, ^6^ Symptoms of parkinsonism are typically bilateral and symmetric without prominent resting tremor. They may manifest within a few days after initiation of neuroleptic therapy and 90% of cases emerge by 3 months.^2^, ^7^ The risk of DIP with different agents varies widely.^2^, ^8^ Postsynaptic D2 receptor block by neuroleptics is closely linked to antipsychotic effect.^2^, ^9^ Production of extrapyramidal symptoms (EPS) is a requirement to classify conventional neuroleptics.^9^ More recent studies indicate that D2 receptor affinity is not necessary for the antipsychotic effect and the production of EPS is not necessary for that benefit. Antipsychotic drugs with low D2 receptor affinity became known as “atypical” antipsychotics, of which clozapine is an example.^9^ With more widespread use of atypical antipsychotic drugs, it is anticipated that the incidence of DIP would decline, though definite evidence for that is not available so far.

After discontinuing the causal agent, persistence, worsening, or symptom resolution followed by reemergence of parkinsonian symptoms is each interpreted as evidence of underlying preclinical Parkinson's disease (PD) being unmasked by DA blockers. Old age, dementia and family history of PD are reported as risk factors for DIP.^1^, ^8^ Autopsy studies have confirmed that some patients who recover from DIP after discontinuing the offending drug have pathological findings characteristic of preclinical PD.^10^, ^11^ The motor features of DIP and PD are similar,^8^ and clinical distinction is not always possible. Single‐photon emission computed tomography (SPECT) imaging with FP‐CIT has been a useful tool to identify preclinical PD, which may be unmasked by drugs. There is reduced dopamine transporter (DAT) uptake in PD and in preclinical degenerative PD, but the uptake is normal in pure DIP.^12^, ^13^

In spite of the large number of DIP cases observed in clinical practice, very few autopsies have been reported in such cases.^10^, ^11^ We report on a detailed clinical and pathologic study in 7 DIP patients.

## Patients and Methods

The Movement Disorders Clinic Saskatchewan (MDCS) has operated continuously since 1968. Details have been reported previously.^14^ All MDCS patients are offered brain autopsy at no cost to the family. Patients are followed by movement disorders specialists (A.H.R., A.R.) at the MDCS at 6‐ to 12‐month intervals. Information on sex, age of symptom onset, past history, use of neuroleptics and other drugs, family history, use of antiparkinson drugs, medication benefits, and adverse effects is obtained from patients/caregivers prospectively. Severity of bradykinesia, rigidity, and tremor as per the Webster scale^15^ and, more recently, by the UPDRS^16^ and global disability using the H & Y scale are assessed at each clinic visit.^14^, ^16^, ^17^ Between clinic visits, the neurologists are available for patient management.

Autopsy is performed after written consent from the next of kin. Autopsy consent is approved by the Saskatoon Health Region, and approval for research on the autopsied brains is granted by the University of Saskatchewan Ethics Board. Immediately after autopsy, the brain is divided at midline. One half of the brain is fixed in formalin and examined histologically by a neuropathologist. The other half is frozen at −80°C for future studies. Contemporary protocols for PD pathology were followed—using the commercially available stains at our institution, including silver stain, ubiquitin, tau, and alpha‐synuclein (α‐Syn) immunostains. A complete neuropathology report is issued on each case and shared with the family.

## Results

Only those cases of DIP who came to autopsy between 1970 ando 2014 were considered for this study. Of the total of 505 autopsies on patients observed in the MDCS, 7 such cases were identified.

### Report of Cases

#### Case 1

A 65‐year‐old man with a long history of bipolar disorder (BPD) was treated with chlorpromazine 75 mg three times daily (TID) since age 41 and lithium 300 mg TID since around age 50. At age 60, he noticed resting tremor in the left upper limb (UL), which worsened over the next 5 years and involved the right side. A trial on benztropine by the family physician produced confusion and hallucinations. Examination at the MDCS at age 65 revealed bilateral UL action tremor, more pronounced on the left, bilateral UL rigidity and bradykinesia. Diagnosis of parkinsonism, stage 2,^16^, ^17^ secondary to neuroleptic use was made. Because of asymmetrical parkinsonism findings, it was considered that he may have underlying PD pathology unmasked by neuroleptics, he was started on selegiline 5 mg once‐daily. It made his “stomach sour” and was therefore discontinued. He was switched to trihexyphenidyl 2 mg TID 2 weeks before his next visit 6 months later when his parkinsonism was essentially unchanged at stage 2 disability.^17^ He continued with chlorpromazine (though at a reduced dose of 50 mg TID), lithium, and trihexyphenidyl and was followed at the MDCS for 5 years. At age 69, orofacial tardive dyskinesias were noted and his wife noted that his memory was not good—he was on trihexyphenidyl 2 mg four times daily (QID), and it was recommended that the dose be reduced. At his last visit to the MDCS at age 70, his parkinsonism was at H & Y stage 2.^16^, ^17^ Toward the end, he was in a nursing home and his medications included quetiapine and lithium; he was no longer on trihexyphenidyl or chlorpromazine. He died at age 85.

On autopsy, the brain weighed 1,410 g and was grossly normal. There were neurofibrillary tangles throughout the brain and amyloid deposits in the hippocampus. There was no loss of pigmented neurons in the SN or in the locus ceruleus (LC). α‐Syn staining was negative. The final pathological diagnosis was mild Alzheimer's disease and mild cortical amyloid angiopathy.

#### Case 2

He had been on long‐term treatment with metoclopramide 10 mg twice‐daily (BID) for reflux owing to a large hiatus hernia. He had onset of gait difficulty at age 78 followed by gait initiation failure, gait freezing, and bilateral UL resting tremor. The metoclopramide dose was reduced to once‐daily. He was started on levodopa/carbidopa 100/25 mg 1 tablet BID 6 weeks before first evaluation at the MDCS, with reported improvement in tremor. On examination at age 83, he had bilateral UL rigidity, right side worse, definite bradykinesia in all four limbs, and UL postural and kinetic tremors. He had mild orofacial chewing movements intermittently. He had a flexed posture and was unable to stand from sitting position without assistance; gait was wide based and shuffling with minimal arm swing. He was diagnosed with PD, H & Y stage 4,^16^, ^17^ and the l‐dopa/carbidopa 100/25 mg was increased to 1 tablet QID, with a final maintenance dose of 2 tablets five times daily. There was some improvement of symptoms, though he remained at H & Y stage 4. Metoclopramide was continued by the family physician and the dose increased to 10 mg TID before his death at age 85.

At autopsy, the brain weighed 1,680 g and was grossly normal except for a hemorrhagic infarct in the right posterior angular gyrus. There were rare cerebral neurofibrillary tangles, but no loss of pigmented neurons in the SN or LC. There were no pathological findings consistent with clinical features of parkinsonism. α‐Syn staining was negative.

#### Case 3

At age 69, he was admitted to local hospital for confusion and treated with lorazepam 0.5 to 1.0 mg up to six times daily as needed (PRN) and risperidone 0.5 mg TID. He was discharged to a nursing home on those drugs. One month later, he was admitted to the Royal University Hospital Saskatoon for confusion and personality change. Clinical diagnosis was encephalopathy. He was investigated extensively. The peripheral blood counts and electrolytes were normal. Multiple electroencephalograms were normal and nerve conduction study was normal. Cerebral angiograms were normal; notably, there was no evidence of vessel occlusion, aneurysm, malformation, or other evidence of vasculitis. Carotid doppler was normal. CT of the brain revealed mild cerebral atrophy and an ill‐defined hypodensity in the left corona radiata. MRI of the brain, MR diffusion study, and MRA of the head revealed diffuse cerebral and cerebellar atrophy and periventricular and subcortical foci of T2 hyperintensity and patchy T2 signal in the left pons. Regional cerebral blood perfusion SPECT study revealed no abnormality.

Cerebrospinal fluid (CSF) was normal for proteins, glucose, and white cell count. Polymerase chain reaction for herpes simplex was negative. CSF culture revealed no organisms. CSF was faintly positive for 14‐3‐3 proteins by immunoblotting. Definite diagnosis for the cause of encephalopathy was not made. He was discharged to the nursing home and was continued on risperidone 0.5 mg TID. Four months later, his wife noted that he was tremulous, had shuffling gait, and visual hallucinations. He could walk less than half a block alone, provided somebody was with him, but after that he needed active support. Examination at that time revealed, mild but definite bradykinesia in all four limbs, bilateral UL mild resting tremor, bilateral kinetic and postural tremor, bilateral mild rigidity, and flexed posture with slow shuffling gait. His Mini–Mental Status Examination (MMSE) score was 21/30. He was diagnosed as having parkinsonism and dementia, likely Lewy body dementia given his visual hallucinations and variable cognition and behavior. His disability was rated at stage 4.0 H & Y.^17^ He was treated with rivastigmine 1.5 mg once‐daily (OD) for 1 week and then increased to BID. It was recommended that the risperidone be changed to quetiapine; however, that was not pursued by the family physician and he was continued on risperidone. There was no symptomatic improvement on rivastigmine. He was reevaluated at the MDCS 6 months later. His MMSE was 18/30 and H & Y stage was 5. He was incontinent of urine and sometimes stool. His tremor had become worse and hallucinations were “too bad” to describe. l‐dopa/carbidopa 100/25 mg OD was initiated. It was increased over 1 week to 1 tablet TID and rivastigmine dose was recommended to increase to 3 mg TID. At his final visit to the MDCS nearly 8 months later on his 71st birthday, his condition had declined further. By then, rivastigmine had been stopped. At that time, he was recommended to increase the l‐dopa/carbidopa 100/25 mg to 1.5 tablets TID. There was no symptomatic benefit. He continued to decline over the next 5 years while in a nursing home. The risperidone was continued until his death at age 75.

At autopsy, the brain weighed 1,380 g. Microscopically there was a moderate loss of pigmented neurons in the LC and a mild loss in the SN. α‐Syn immunostaining was negative, as was that for ubiquitin, TDP‐43, and FUS, thus ruling out common forms of tau‐negative degenerations. Tau immunostaining revealed changes consistent with an Alzheimer's disease neuropathological score of “low” according to 2012 National Institute on Aging‐Alzheimer's Association guidelines.^18^ Thus, although not ruled out, a definite diagnosis of Alzheimer's disease could not be made, and the cause for his dementia could not be established.

#### Case 4

He was seen at age 55 years in our clinic in May 1974. He had a long history of depression, anxiety, and insomnia. He was hospitalized under psychiatry at age 42 and was noted to have obvious tremor. He received electroconvulsive treatment and was started on chlorpromazine and benztropine. Chlorpromazine was continued until his death. He had onset of UL tremor at age 23, and at age 50, was diagnosed as PD by the family physician and treated with l‐dopa. That was discontinued because of severe nausea, but had no symptomatic benefit. Examination at the MDCS at age 55 revealed a slow head tremor with both vertical and horizontal components, moderate bilateral UL resting tremor and more pronounced action tremor, and mild bradykinesia and rigidity in both ULs and unstable posture—stage 3 H & Y.^17^ He was diagnosed as having essential tremor and parkinsonism, secondary to antipsychotics. He was not willing to try antiparkinson drugs. He died of myocardial infarction at age 58.

On autopsy, the brain weighed 1,400 g and was grossly normal. There was no loss of pigmentation of SN or other pathology consistent with parkinsonism noted grossly or microscopically. Brain histology otherwise was normal.

#### Case 5

At age 61, he had noticed bilateral UL tremor during writing, which became worse at age 65 subsequent to the death of his wife. He was hospitalized for depression and was treated with electroconvulsive therapy and started on phenothiazine at age 66. Examination at the MDCS 2 months later revealed moderate bilateral UL postural and action tremor; the right side was worse. In addition, there was right UL resting tremor mostly at the thumb, and mild bradykinesia and rigidity in the left UL. Because his primary problem was tremor, he was started on propranolol. Three months later, propranolol was discontinued because he did not think it was effective (though objectively the tremor appeared improved). At that visit, he had UL postural, action, and resting tremor and questionable rigidity, but no definite bradykinesia. Diagnosis of PD was made. He died at age 67 of myocardial infarction.

On autopsy, the brain weighed 1,350 g. There was marked cerebral arteriosclerosis and enlarged perivascular spaces, in the basal ganglia. The brain was otherwise grossly and microscopically normal. There was no loss of SN or LC neurons.

#### Case 6

He was hospitalized at age 69 with a 2‐week history of confusion, agitation, and combative tendencies after he had hit his head against the stairs. He was treated with intramuscular chlorpromazine 50 mg TID, with additional doses given as necessary. Six days later, he was noted to have bilateral, small‐amplitude resting tremor in the upper limbs and moderate generalized rigidity and bradykinesia. A diagnosis of NIP was made. The following day, he received chlorpromazine 75 mg intramuscular and 2 mg of benztropine orally. Both drugs were discontinued the next day. The parkinsonian manifestations subsided completely during the next 7 days. He developed respiratory symptoms and he died 1 month later.

At autopsy, pulmonary embolism and bronchopneumonia were confirmed as the cause of death. The brain weighed 1,500 g and was grossly normal. Moderate loss of SN pigmented nerve cells, gliosis, and frequent intracytoplasmic Lewy bodies were found. Lewy bodies were also observed in the LC. DA and homovanillic acid levels were moderately reduced in the caudate and the putamen. The final diagnosis was preclinical PD unmasked by neuroleptics.^10^

#### Case 7

He was known to have bipolar disorder and was treated with haloperidol 2 mg TID and benztropine for 3 months, before his hospitalization in May 1977 at age 59. On examination, he had moderate generalized rigidity, bradykinesia, and resting tremor in both ULs. The neuroleptics and benztropine were discontinued. The parkinsonian features resolved over the next 2 weeks. He died suddenly 2 months later at age 59.

At autopsy, acute pulmonary edema was detected. Histological examination of the brain revealed slight loss of SN pigmented cells and numerous intracytoplasmic Lewy bodies (LB). Biochemical assay of the striatum showed no measurable reduction of DA levels.^10^

## Discussion

Striatal DA deficiency is well recognized as the primary biochemical abnormality in PD.^19^ Histologically, marked SN neuronal loss is characteristic of PD.^20^, ^21^ DIP shares clinical features with PD,^2^ but there are no known histological changes in the DIP brains. PET studies show that blockage of 75% to 80% postsynaptic D_2_ receptors results in motor features of parkinsonism.^22^ Whereas hundreds of autopsied cases of PD have been reported in the literature, fewer than 20 DIP autopsies have been reported thus far.^10^, ^11^

DIP is produced by several different classes of drugs. Whereas the D_2_ receptor blockage is known in the NIP,^2^, ^23^ the mechanism of DIP owing to other drugs is not fully understood.^1^ By definition, the older neuroleptics always produce EPS.^9^, ^23^ Other drugs with no significant D_2_ receptor affinity also produce DIP, indicating that DA receptor blockage is not the sole mechanism of DIP.

A “double hit” hypothesis has been proposed, with DA‐blocking agents also producing neurotoxicity and pathological changes of PD, though this has not been proven.^23^ Our data do not support neuroleptic‐induced SN neurotoxicity as measured by standard neuropathology. It is possible that there are some changes with chronic neuroleptic use, which compromise SN neuronal function without causing neuronal death, resulting in persistent parkinsonism.

Nonmotor functions were not specifically identified in our cases; however, recent work suggests that nonmotor features, such as disordered olfaction and cardiac denervation, may predict those who will go on to develop persistent PS.^24^, ^25^, ^26^ Autopsy verification has, however, not been performed in these cases.

Our cases broadly represent three subgroups: Cases 6 and 7 had underlying PD pathology and were previously reported on in detail.^10^ Cases 1, 2, 4, and 5 had no pathological changes that may produce parkinsonism or predispose to neuroleptic‐induced Parkinson syndrome (PS). Case 3 had mild SN neuronal loss, but no LB inclusions. Some SN loss is observed with normal aging,^27^, ^28^ but the subregional pattern of neuronal loss is different than in the PD.^28^ The exact threshold of SN neuronal loss to manifest parkinsonian motor features has not been established. However, slight to moderate loss on standard neuropathology study, as observed in our cases 6 and 7, does not produce clinical features of parkinsonism. Autopsy studies report between 65% and 80% SN neuronal loss in PD cases.^29^ The mild neuronal loss in case 3, we believe, is not the basis of parkinsonism, though it could have predisposed this patient to NIP.

In addition to the clinical features of PS, two of our cases (1 and 2) had tardive dyskinesia (TD). That combination is common in patients on DA receptor‐blocking agents.^1^ It is estimated that there is a 2‐fold higher risk of TD in DIP cases.^30^ Concurrent use of anticholinergics in neuroleptic‐treated cases is believed to increase the risk of TD.^30^ Case 1 was concurrently on anticholinergic medication.

Bower and colleagues^11^ reported on eight cases with clinical diagnosis of DIP. In three of those, the PS resolved after discontinuing neuroleptics. One of those three had LB pathology and one had vascular pathology. These cases resemble our cases 3, 6, and 7. In five of their cases that were continued on the neuroleptics until death, four had other identifiable pathology.^11^ Thus, two cases in that series—one with reversible DIP and one with DIP on continued neuroleptic use—had no identifiable pathological process, as in four of our cases (1, 2, 4, and 5).

Together, these observations indicate that even after prolonged use of neuroleptics, there are no histological changes including no SN neuronal loss. This supports the observations that DIP in such cases is primarily the result of DA receptor blockage. Cessation of PS features on discontinuing the offending drug indicates that the DA receptor blockage is reversible. Given that all DIP cases do not resolve after discontinuing the DA‐blocking drugs, DA receptor change may be irreversible in some cases or there are other as yet unknown changes that account for the ongoing parkinsonism. Further studies are needed to identify the pathophysiology in such cases.

Given that the D_2_ receptor block on the neuroleptics occurs bilaterally, it is expected that NIP would have symmetrical clinical features. Asymmetrical parkinsonism manifestations favor a PD diagnosis,^20^ and imaging studies indicate that DIP cases with asymmetrical clinical features are more likely to have underlying degenerative PD.^13^ Up to 44% of DIP cases have functional imaging findings indicative of underlying degenerative PD.^23^ However, those have not been verified pathologically. None of our patients had DAT or PET scan. In one study,^7^ 54% and in another study^31^ 30% of the DIP cases had asymmetrical clinical features. Three of our cases had asymmetrical findings, but none had underlying SN or other pathology. On the other hand, the two preclinical PD cases (6 and 7) unmasked by neuroleptics had a symmetrical parkinsonian finding. These observations indicate that asymmetrical parkinsonism is a part of DIP spectrum.

Rest tremor, which is a characteristic of PD, but is considered uncommon in DIP, was observed in all seven of our NIP cases at some time during the course.

Our data do not permit detailed comments on treatment of DIP cases. It has been a common practice to use anticholinergic prophylaxis with the prescription of neuroleptics.^8^ Anticholinergics are not without risk, especially so in the elderly. They can produce memory loss, confusion, dry mouth, urinary retention, and aggravate glaucoma. Prophylactic use of anticholinergic drugs may also increase the risk of TD.^30^ A large proportion of individuals on neuroleptics do not develop PS.^8^ Therefore, prophylactic use of anticholinergics in all cases treated with neuroleptic is not justified.

The effective symptomatic drugs for DIP reported in the literature are the anticholinergics^32^ and amantadine.^33^ Though the efficacy is similar, the adverse effects profile favors amantadine.^33^ Some symptomatic benefit has been reported on l‐dopa, but compared to PD cases, the response is inconsistent and modest at best.^7^

## Author Roles

(1) Research Project: A. Conception, B. Organization, C. Execution; (2) Statistical Analysis: A. Design, B. Execution, C. Review and Critique; (3) Manuscript Preparation: A. Writing of the First Draft, B. Review and Critique.

U.A.S.: 1A, 1B, 3A, 3B

A.H.R.: 1A, 1B, 1C, 3A

C.A.R.: 1C, 3B

A.R.: 1B, 1C, 3A, 3B

## Financial Disclosures

A.H.R. received an honorarium from Parkinson Society Canada. A.R. received research support from the Regina Curling Classic, Greystone Classic for Parkinson's, Inc. and the Dr. Ali Rajput Endowment for Parkinson's Disease and Movement Disorders.
